# Deaths and disability-adjusted life years of hypertension in China, South Korea, and Japan: A trend over the past 29 years

**DOI:** 10.3389/fcvm.2023.1080682

**Published:** 2023-03-15

**Authors:** Yan Qiu, Junzhuang Ma, Jiahong Zhu, Ying Liu, Wen Ren, Shuaishuai Zhang, Jingjing Ren

**Affiliations:** ^1^ Department of General Practice, The First Affiliated Hospital, School of Medicine, Zhejiang University, Hangzhou, China; ^2^ Department of General Practice, The First Division Hospital of Xinjiang Production and Construction Group, Aksu, China

**Keywords:** blood pressure, population, mortality, DALYs, burden

## Abstract

**Background:**

Hypertension has been confirmed as an independent risk factor for cardiovascular disease and death. Few data were analyzed on deaths and disability-adjusted life years (DALYs) caused by hypertension in East Asia. We aimed to provide an overview of burden attributable to high blood pressure in China in the past 29 years, compared with those in Japan and South Korea.

**Methods:**

Data were collected from the 2019 Global Burden of Disease study on diseases due to high systolic blood pressure (SBP). We retrieved the age-standardized mortality rate (ASMR) and DALYs rate (ASDR) by gender, age, location, and sociodemographic index. The death and DALY trends were evaluated by estimated annual percentage change, with 95% confidence interval.

**Findings:**

Considerable differences were detected in the diseases attributable to high SBP in China, Japan, and South Korea. In 2019, the ASMR and ASDR of diseases due to high SBP in China were 153.34 (126.19, 182.49) per 100,000 population and 2,844.27 (2,391.91, 3,321.12) per 100,000 population, respectively, which was about 3.50-fold of those in another two countries. The elders and males had higher ASMR and ASDR in the three countries. Between 1990 and 2019, the declining trends were less pronounced in China for both the deaths and DALYs.

**Conclusions:**

The deaths and DALYs due to hypertension declined in China, Japan, and South Korea in the past 29 years, with China having the greatest burden.

## Introduction

Hypertension has been confirmed as an independent risk factor for CVDs and deaths (1–3). According to the Global Burden of Disease (GBD) Study, 10.8 million deaths attributed to high systolic blood pressure (SBP) was the leading level 2 risk factor globally, which accounted for 19.2% of all deaths in 2019 (4). High SBP remains a public health problem globally and its attributable disease burden varies among regions (5). Disease burden attributable to high SBP has been conducted in the world with several updates (5–8). However, there were few studies on disease burden attributable to high SBP in East Asia.

Being neighboring countries, China, South Korea, and Japan are located in East Asia with similar culture and genetic backgrounds. However, these three countries had different socioeconomic development. China is behind South Korea and Japan in urbanization and industrialization (9), which may result in variations in some disease burden. Therefore, we aimed to determine the deaths and disability-adjusted life years (DALYs) caused by high SBP in China, compared with Japan and South Korea.

## Materials and methods

### Data sources

The GBD 2019 study was a comprehensive and updated data source, which included 87 risk factors, 369 diseases, and injuries across 204 countries and territories (10). In China, two primary sources of data were surveillance data from the China Disease Surveillance Points system and vital registration data collected by the Chinese Center for Disease Control and Prevention. In this study, we used GBD 2019 to retrieve data on deaths and DALYs of diseases caused by high SBP. We also retrieved the age-standardized rates (ASR) of mortality (ASMR) and DALY (ASDR) of diseases attributed to high SBP in China, South Korea, and Japan from 1990 to 2019. Global cases and ASRs were also collected for analysis. Here, high SBP was defined as individual with a theoretical minimum risk exposure level of over 115 mmHg in the GBD 2019 (4).

All data in this analysis were de-identified and publicly available at the IHME website (http://ghdx.healthdata.org/gbd-results-tool). Hence, the study was reviewed and waived of informed consent by the Ethics Committee of the First Affiliated Hospital, School of Medicine, Zhejiang University, China. The study complied with the Guidelines for Accurate and Transparent Health Estimates Reporting recommendations (11).

### Case definition

Diseases attributable to high SBP were diagnosed and defined in accordance with the 10th version of International Classification of Diseases (ICD-10) and the WHO clinical criteria. In this study, diseases due to high SBP included level 4 causes (10). Level 1 cause was noncommunicable diseases (NCDs). Level 2 causes included cardiovascular diseases (CVDs), diabetes, and kidney diseases. Level 3 included 12 diseases, and the list of ICD-10 for diseases attributable to high SBP could be seen in the Supplementary Table S1.

### Sociodemographic Index

Sociodemographic index (SDI) ranged from 0 to 100 in GBD 2019, which comprised of low, low–middle, middle, high–middle, and high SDI countries in GBD studies. It was calculated from the total fertility rate in women younger than 25 years, lag-distributed income per capita, and mean education for individuals aged 15 years and older (12). The relation of SDI and ASR was determined with the Pearson correlation coefficient, and the expected relation for countries was fitted with a Loess smoother between 1990 and 2019.

### Summary exposure value

The definition of summary exposure value (SEV) could be seen in a previous study (4).

It was a value on the scale of 0–100, meaning everyone in the population had minimum risk with 0 to maximum risk with 100.

### Population attributable fraction

The population attributable fraction (PAF) is the proportion of reduced deaths of disease in a certain population, if exposure to a certain risk factor is decremented to the theoretical minimum exposure level in the population. In GBD studies, the attribution of deaths and DALYs to high SBP was calculated by multiplying PAFs for a given age–sex–location–year. Further details regarding the estimation of high SBP and its attributable burden have been provided in a previous study (4).

### Statistical analysis

All the statistical analyses, plots, and numbers created in this study were performed by R version 4.2.1 and RStudio version 2022.07.0 + 548 (https://www.rstudio.com/, © 2009–2022 RStudio, Inc.). We reported all case numbers and their corresponding ASRs per 100,000 people with 95% uncertainty intervals (UIs). The ASRs were calculated by the GBD world population age standard. PAF values were reported with a 95% confidence interval (CI).

The estimated annual percentage changes (EAPCs) were calculated to evaluate the deaths and DALYs trends, with 95% CI for EAPCs. The EAPC was calculated as [(Exp (*β*) − 1)] × 100, and the 95% CI was calculated by a linear model. In the linear regression model, the natural logarithm of ASR was calculated as *y* = *α *+ *βx *+ *ε*, where *y* = ln(ASR) and *x* = calendar year (13). When the estimated value and lower 95% CI of the EAPC were both >0, the ASRs were considered to be increasing; when the estimated value and upper 95% CI of the EAPC were both <0, the ASRs were considered to be decreasing. Otherwise, the ASR was considered to be stable. The *p* value of 0.05 was determined as significant.

## Results

### Disease burden and trend attributable to high SBP

Globally, the ASMR and ASDR of diseases due to high SBP were 138.88 (95% UI: 121.25, 155.73) per 100,000 population and 2,885.57 (95% UI: 2,580.75, 3,201.05) per 100,000 population in 2019, respectively (Table 1). Decreasing trends were detected from 1990 to 2019, with an EAPC of −1.32% (95% CI: −1.36%, −1.27%) for deaths and −1.17% (95% CI: −1.22%, −1.13%) for DALYs.

**Table 1:** The ASMR, ASDR, and EAPC of disease due to high SBP between 1990 and 2019 by location and sex.

Location	Sex	1990 (95% UI)	2019 (95% UI)	EAPC (95% CI)
**Deaths**				
Global	Both	197.87 (174.93, 220.93)	138.88 (121.25, 155.73)	−1.32 (−1.36, −1.27)^a^
	Male	218.54 (192.84, 243.49)	160.13 (138.91, 180.79)	−1.11 (−1.14, −1.08)^a^
	Female	178.86 (154.91, 201.06)	119.66 (102.33, 136.86)	−1.53 (−1.59, −1.47)^a^
China	Both	190.99 (159.6, 226.22)	153.34 (126.19, 182.49)	−0.47 (−0.60, −0.35)^a^
	Male	212.79 (171.5, 258.83)	197.23 (155.39, 239.98)	0.16 (0, 0.32)^a^
	Female	175.75 (137.92, 216.61)	122.88 (93.31, 154.81)	−1.02 (−1.14, −0.90)^a^
Japan	Both	118.05 (101.21, 131.9)	42.01 (34.09, 48.61)	−3.74 (−3.93, −3.55)^a^
	Male	141.34 (123.98, 156.93)	56.49 (47.43, 64.45)	−3.29 (−3.41, −3.16)^a^
	Female	100.34 (84.16, 113.32)	29.69 (22.04, 36.3)	−4.48 (−4.74, −4.23)^a^
South Korea	Both	213.68 (181.67, 242.88)	45.15 (35.43, 55.71)	−6.03 (−6.29, −5.76)^a^
	Male	249.09 (203.36, 284.92)	49.13 (39.17, 61.91)	−6.08 (−6.33, −5.82)^a^
	Female	188.74 (156.82, 217.79)	40.16 (29.86, 50.58)	−6.17 (−6.49, −5.84)^a^
**DALYs**				
Global	Both	3,953.92 (3,557.53, 4,359.13)	2,885.57 (2,580.75, 3,201.05)	−1.17 (−1.22, −1.13)^a^
	Male	4,538.11 (4,060.32, 5,008.08)	3,448.86 (3,060.06, 3,837.69)	−0.99 (−1.03, −0.95)^a^
	Female	3,403.35 (3,025.28, 3,766.52)	2,354.72 (2,075.57, 2,634.68)	−1.41 (−1.47, −1.36)^a^
China	Both	3,672.42 (3,072.59, 4,301.4)	2,844.27 (2,391.91, 3,321.12)	−0.66 (−0.75, −0.56)^a^
	Male	4,053.6 (3,258.07, 4,913.82)	3,569.96 (2,837.6, 4,370.56)	−0.11 (−0.23, 0.01)^a^
	Female	3,337.46 (2,635.55, 4,083.3)	2,224.66 (1,727.63, 2,742.72)	−1.26 (−1.37, −1.16)^a^
Japan	Both	2,139.39 (1,919.54, 2,336.61)	924.25 (806.02, 1,040.86)	−3.05 (−3.20, −2.91)^a^
	Male	2,683.81 (2,442.05, 2,910.87)	1,264.8 (1,117.59, 1,417.8)	−2.70 (−2.79, −2.60)^a^
	Female	1,685.79 (1,484.28, 1,857.2)	609.07 (504.92, 718.63)	−3.79 (−4.01, −3.56)^a^
South Korea	Both	4,062.22 (3,510.71, 4,545.92)	796.35 (663.09, 958.39)	−6.30 (−6.58, −6.03)^a^
	Male	4,934.05 (4,181.35, 5,540.78)	950.87 (779.03, 1,185.22)	−6.12 (−6.38, −5.87)^a^
	Female	3,367.1 (2,896.2, 3,791.37)	634.03 (510.46, 766.81)	−6.69 (−7.04, −6.35)^a^

ASMR, age-standardized mortality rate; ASDR, age-standardized DALY rate; DALYs, disability-adjusted life years; EAPC, estimated annual percentage change; UI, uncertainty interval; CI, confidence interval.

^a^ Changes that are statistically significant.

Considerable heterogeneity was observed in the deaths and DALYs due to high SBP in China, Japan, and South Korea. In 2019, the ASMR and ASDR of disease due to high SBP in China were 153.34 (126.19, 182.49) per 100,000 population and 2,844.27 (2,391.91, 3,321.12) per 100,000 population, respectively, which was about 3.50-fold of the age-standardized rates in Japan and South Korea. Although the ASMR and ASDR decreased in these three countries from 1990 to 2019, the decline in Japan and South Korea was more pronounced than that in China. The EAPC of ASMR was −0.47% (95% CI: −0.60%, −0.35) in China, about one-twelfth and one-seventh of that in South Korea and Japan, respectively. The EAPC of ASDR was −0.66% (95% CI: −0.75%, −0.56%) in China, approximately one-tenth and one-fifth of that in South Korea and Japan.

### Disease burden attributable to high SBP by sex and age

**Figure 1:**
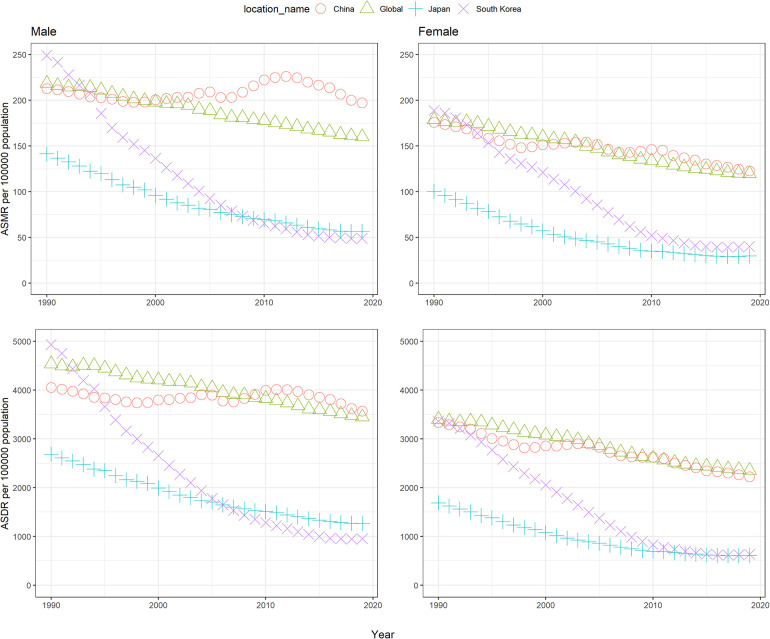
The age-standardized rates of mortality and DALYs attributable to high systolic blood pressure from 1990 to 2019 for global, China, Japan, and South Korea. ASMR: age-standardized mortality rate, ASDR: age-standardized DALY rate..

Men had higher ASMR and ASDR of disease due to high SBP than women in these three countries (Table 1, Figure 1). Remarkable differences were observed for male-to-female sex ratio of the ASMR and ASDR of disease due to high SBP across countries (Supplementary Figure S1). Japan had the highest sex ratio of the ASMR of disease due to high SBP, followed by China. Both countries had a higher sex ratio than the world, and the sex ratios had been rising since 1990 and began to decline after 2015. In South Korea, the sex ratio was always lower than the global level since 1995, showing a “binominal” shape from 1990 to 2019.

**Figure 2:**
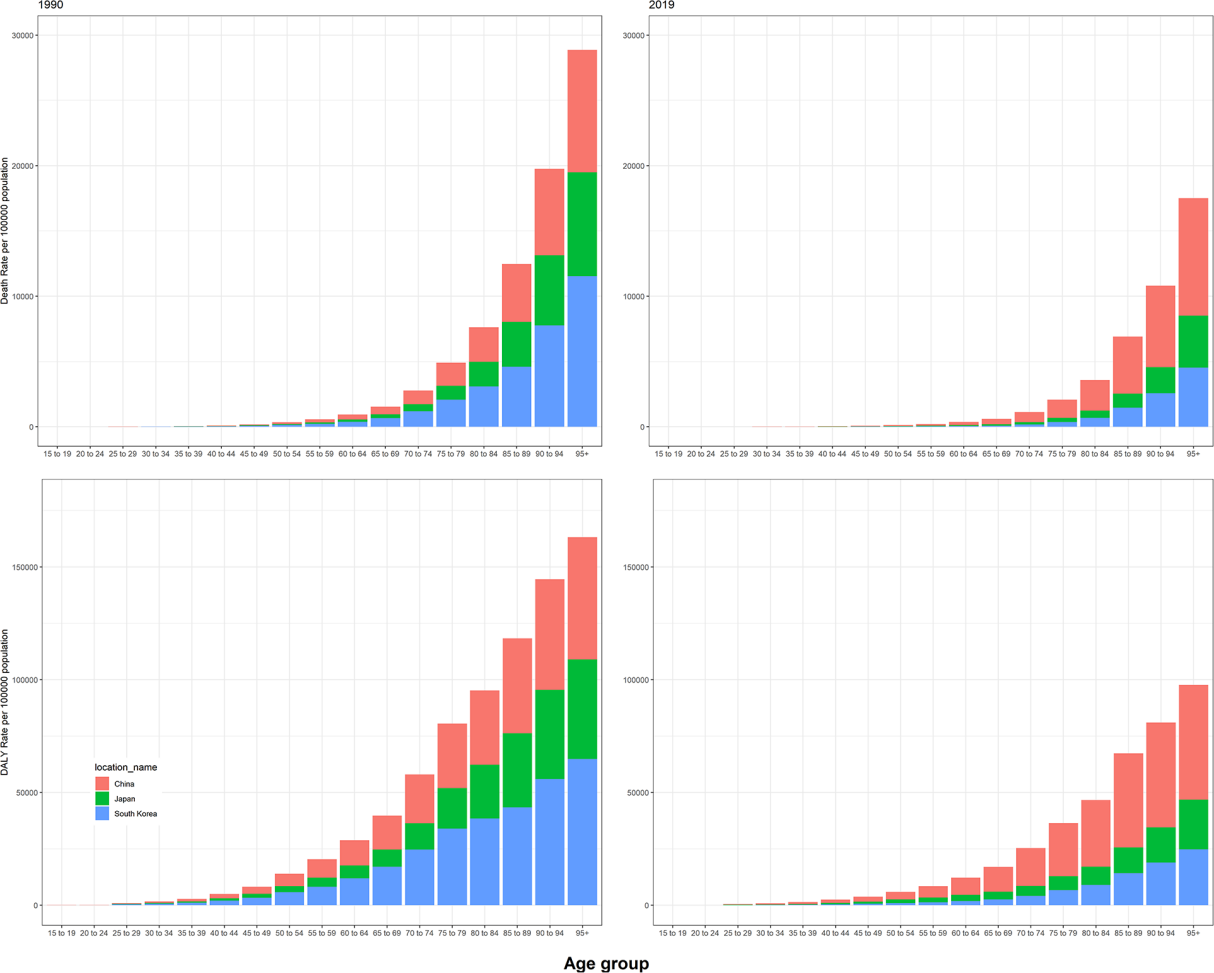
Age-specific rates of mortality and DALYs attributable to high systolic blood pressure in 1990 and 2019 in China, Japan, and South Korea.

Figure 2 showed the age-specific rates of deaths and DALYs due to high SBP in China, Japan, and South Korea. Whatever the year, both the rates of deaths and DALYs presented an increasing trend and peaked at the 95+ years old group.

### Trends of high SBP SEV

Globally, the age-standardized SEV of high SBP were relatively stable, with an SEV of 27.12 (95% UI: 25.51, 28.87) in 1990 and 27.74 (95% UI: 25.70, 29.72) in 2019 (Supplementary Figure S2). China showed an increasing trend in the SEV of high SBP, increasing from 19.15 (95% UI: 15.48, 23.47) in 1990 to 28.05 (95% UI: 22.89, 34.13) in 2019. The rate was higher than the global level in 2018 and 2019. The SEV of high SBP in South Korea decreased from 22.25 (95% UI: 19.44, 25.11) in 1990 to 14.97 (95% UI: 12.44, 17.73) in 2019. In Japan, the SEV decreased from 36.98 (95% UI: 35.33, 38.74) in 1990 to 24.14 (95% UI: 22.59, 25.77) in 2011 and began to rise slowly after 2011. Additionally, males had higher SEV of high SBP than females, both in South Korea and Japan. Although in China, males had lower SEV of high SBP from 1990 to 2011 and then became higher than females since 2012. In 2019, the SEV of high SBP were nearly at the same level in China and the world, with the SEV values of 28.04 (95% UI: 22.89, 34.13) and 27.74 (95% UI: 25.70, 29.72), respectively.

### Relationship between disease burden due to high SBP and SDI

**Figure 3:**
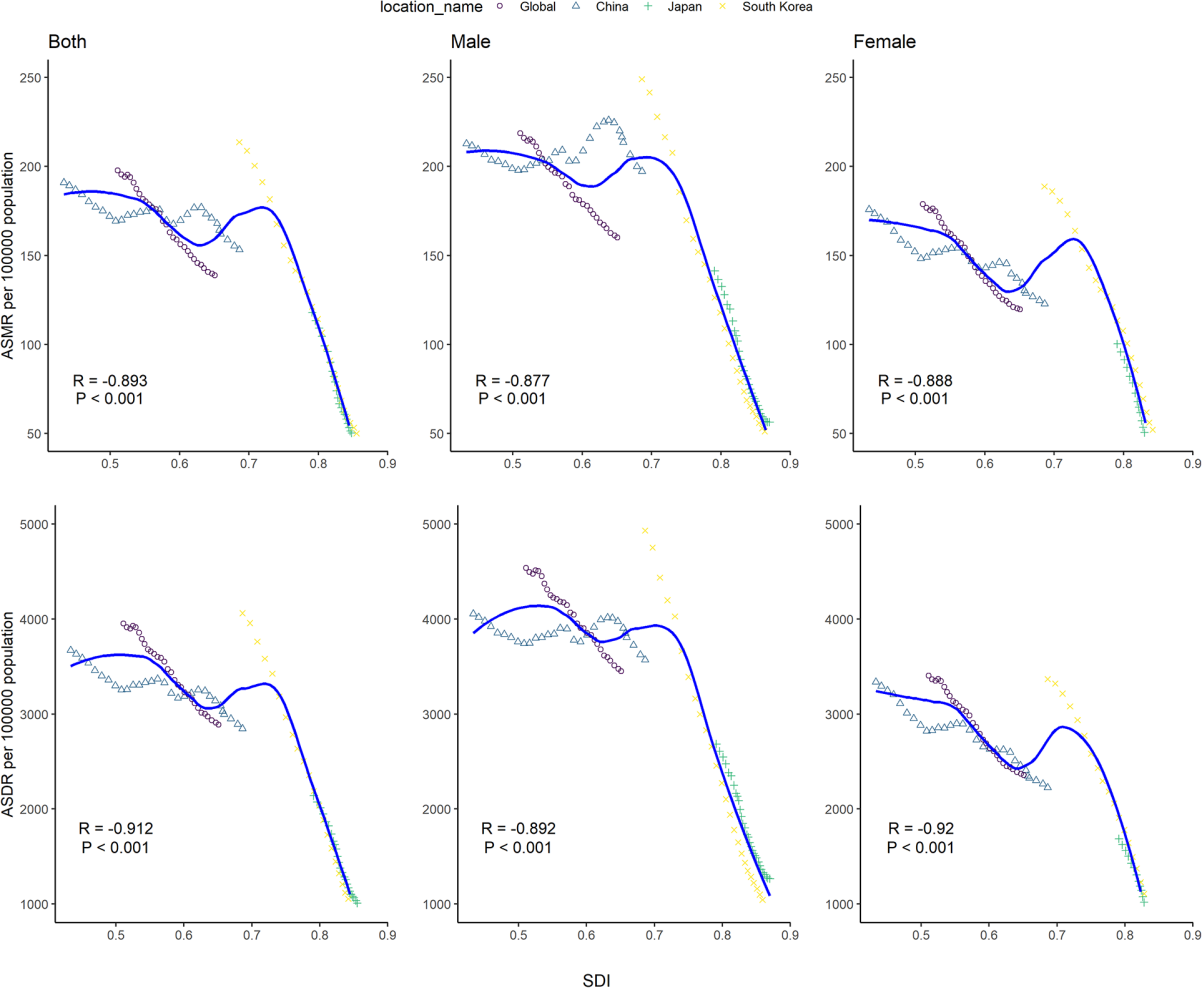
Age-standardized rate of mortality and DALYs of disease attributable to high systolic blood pressure for global, China, Japan, and South Korea, 1990–2019. The solid line shows expected values across the spectrum of the socio-demographic index. The Pearson correlation coefficient and its p-value were denoted. ASMR: age-standardized mortality rate, ASDR: age-standardized DALY rate, SDI: socio-demographic index.

A negative correlation was found in the ASMR and ASDR due to high SBP and SDI (Figure 3). Sex and country had no affect on the relationship. Based on SDI, China and South Korea had slightly higher ASMR of disease due to high SBP than expected for half of the years from 1990 to 2019. In contrast, the ASDR of disease due to high SBP was slightly higher than expected in the world and Japan for most of the years during the same period. In South Korea, the ASMR and ASDR were higher than expected in females, based on their SDI values, while in Japan, the ASMR and ASDR were higher than expected in males, based on their SDI values.

**Figure 4:**
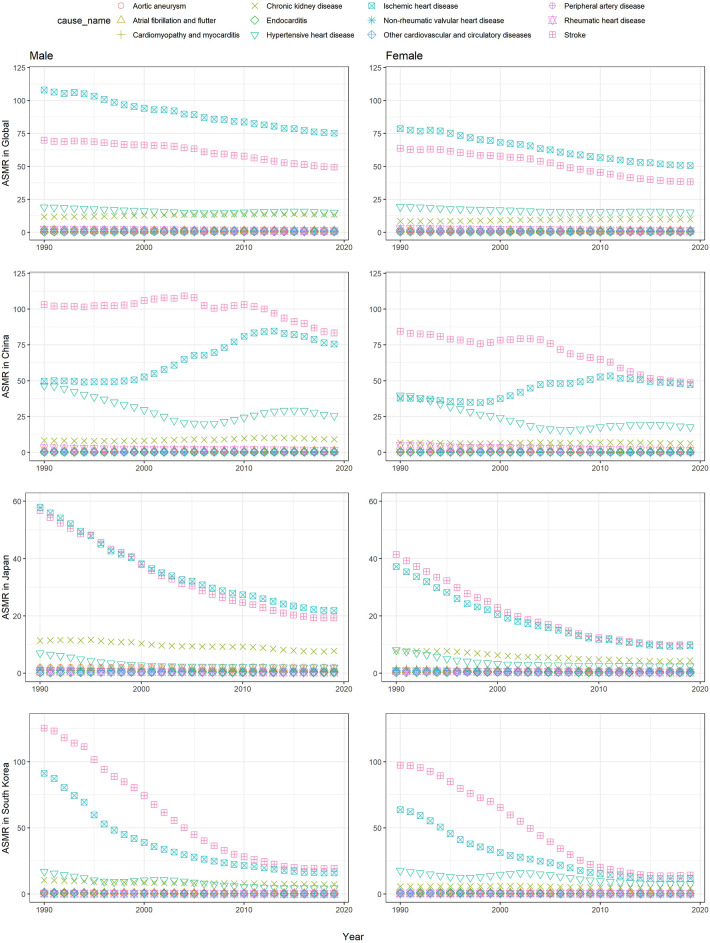
The age-standardized mortality rates of causes attributable to high SBP in China, Japan, and South Korea from 1990 to 2019.

### Burden due to high SBP by causes

In GBD 2019, 12 diseases in level 3 resulted from high SBP, including ischemic heart disease (IHD), hypertensive heart disease (HHD), peripheral artery disease, endocarditis, chronic kidney disease (CHD), cardiomyopathy and myocarditis, non-rheumatic valvular heart disease, rheumatic heart disease, other cardiovascular and circulatory diseases, aortic aneurysm, stroke, and atrial fibrillation and flutter.

From 1990 to 2019, IHD and stroke were the two leading causes of deaths due to high SBP in China, Japan, and South Korea (Figure 4). The downward trends could be observed in the world and Japan. Japan showed a dramatic downward trend with the lowest ASMR of 15.17 (95% UI: 11.35, 18.87) for IHD and 14.23 (95% UI: 11.12, 17.48) per 100,000 population for stroke, respectively, in 2019. Unlike the global situation, IHD dropped from the first to second in rank, and stroke became the top one in China and South Korea. Similar to the global trend, South Korea also witnessed a declining trend, with the ASMR of 16.65 (95% UI: 12.25, 22.48) per 100,000 population for stroke and 13.98 (95% UI: 9.66, 18.84) per 100,000 population for IHD in 2019. In China, the ASMR of stroke remained highly stable and began to decrease since 2010, whereas the ASMR of IHD kept increasing since 2000 and began to decline after 2011. The ASMR of stroke and IHD were similar between 2013 and 2019. In 2019, the rate was 63.20 (95% UI: 4922, 77.65) per 100,000 population and 58.84 (95% UI: 43.28, 74.91) per 100,000 population for stroke and IHD, respectively. In terms of sex, men had a higher ASMR of disease due to high SBP than that of women.

Similar to the deaths, IHD and stroke were the two leading causes of DALYs due to high SBP in China, Japan, and South Korea (Supplementary Figure S3). Japan and South Korea had a declining trend from 1990 to 2019. Slightly different from that of deaths, stroke was always the leading cause of DALYs due to high SBP in China, Japan, and South Korea. China had the highest ASDR of stoke and IHD in 2019, compared with Japan and South Korea.

## Discussion

This study estimated the deaths and DALYs due to high SBP in China, Japan, and South Korea. In 2019, the rates of disease due to high SBP were much higher in China than those in Japan and South Korea. Men had higher ASDR and ASMR of disease due to high SBP than women. Based on GBD 2019 data, the burden in the three countries declined over time with years’ efforts to control blood pressure. However, the decrements varied widely. South Korea had the highest decline, while China had the lowest decline. China should develop and implement more strategies to reduce the high SBP-associated burden.

In this study, IHD and stroke were the leading causes of deaths and DALYs due to high SBP in China, Japan, and South Korea. As to the disease spectrum of deaths and DALYs associated with high SBP, significant differences could be detected in China, Japan, and South Korea. Stroke and IHD were the most common diseases in China and South Korea, but the situation was worse in China. China had relatively slower decreasing trend of age-standardized rates of stroke than South Korea. Unlike South Korea with a sharp declining trend of age-standardized rates of IHD, an increasing trend could be observed in China for the age-standardized rates of IHD.

According to randomized controlled trials, antihypertensive treatment could lower the risk of cardiovascular disease and all-cause mortality (14–16). Correspondingly, the low treatment rate might explain the relative higher burden of cardiovascular disease and death. In Korea, the treatment and control rate of hypertension were 63% and 47% among all adults in 2018, respectively. Among the participants with hypertension who received treatment, the control rate was as high as 73.1% in 2017–2018 (17, 18), while in China, the treatment and control rates of hypertension were 22.9% and 5.7% among adults aged 35–75 years, respectively (19). Even in Northern China, the therapeutic rate, control, and control undertreatment rate of hypertension were 43.3%, 8.6%, and 19.8% among populations aged 35–75 years, respectively (20). The treatment and control rate of hypertension was higher in Korea than in China, with the highest rate in Korea in the world in 2019 (21), calling for Chinese government and health providers to take greater efforts and more comprehensive strategies to prevent further development of disease attributable to high SBP.

Notable variations were also detected for high SBP-attributable disease burden, with higher ASMR and ASDR in China than that in South Korea and Japan. The downward trend of the ASRs of disease attributable to high SBP could be seen with increasing SDI in the three countries. China, being the largest developing country, had much higher SEV due to high SBP than that in Japan and South Korea. Different from the declining trend of age-standardized SEV in South Korea and Japan, an upward trend was observed in China. Consequently, China had the highest deaths and DALYs attributable to high SBP among the three countries.

A dramatically rising trend was observed in rates of deaths and DALYs due to high SBP with age in China, South Korea, and Japan. The rates peaked at the 95+ years old group. The findings were in line with previous observations (22–24). As IHD and stroke were age-related and the leading causes attributable to high SBP in China, Japan, and South Korea, it is of crucial importance to reinforce the prevention of diseases due to high SBP among older adults.

We found that males had higher ASRs of the disease burden attributable to higher SBP than females in all three countries. Previous studies could explain the phenomenon. A study conducted in Korea observed that women had a higher rate of awareness, treatment, and control of NCDs than men (25, 26). Similarly, a cross-sectional survey showed that men often had less health consciousness compared with women in China, Japan, and South Korea (27). Additionally, women had less chance to be exposed to unhealthy eating habits, smoking, drinking, and so on. The different chances for exposure to environmental and social risks might lead to sex disparity due to high SBP (5).

There were indeed limitations in this study. First, it was the availability and high quality of primary data as described in previous GBD studies (4, 5), which might undermine the accuracy and robustness of estimations and cause data bias. Second, the risk curve of high SBP was still assumed as a log-linear relationship in GBD 2019. It would be reassessed in future GBD updates. Third, the estimations may be influenced by different access to SBP testing methods and high SBP-related disease diagnostic technologies (5). Fourth, data for deaths might be underestimated, because it is difficult to distinguish death from high SBP-related causes such as stroke and its comorbidities (28). Fifth, comorbidity of chronic diseases related to high SBP and the combined effects of risk factors remain the issue for further investigation to address. Sixth, uncertainty intervals might be underestimated in regions where data are unavailable on diseases related to high SBP. Hence, more data in remote regions should be collected with high quality for further analysis.

## Conclusions

Our study determined the deaths and DALYs due to high SBP in China, Japan, and South Korea. The burden of diseases due to high SBP declined sharply in Japan and South Korea from 1990 to 2019. However, the burden remained high in China. High blood pressure still poses considerable risk to public health; a variety of approaches and regulations still need to be taken to promote prevention of diseases attributed to high SBP in these countries, especially in China. The findings will help policymakers and physicians to enact plans and policies for blood pressure control and disease prevention.

## Data Availability

Publicly available datasets were analyzed in this study. All data are publicly available from the Global Health Data Exchange query tool (http://ghdx.healthdata.org/gbd-results-tool).
